# Widespread hepatitis B virus genotype G (HBV-G) infection during the early years of the HIV epidemic in the Netherlands among men who have sex with men

**DOI:** 10.1186/s12879-016-1599-7

**Published:** 2016-06-10

**Authors:** Marion Cornelissen, Fokla Zorgdrager, Sylvia M. Bruisten, Margreet Bakker, Ben Berkhout, Antoinette C. van der Kuyl

**Affiliations:** Laboratory of Experimental Virology, Department of Medical Microbiology, Center for Infection and Immunity Amsterdam (CINIMA), Academic Medical Center of the University of Amsterdam, Meibergdreef 15, 1105 AZ Amsterdam, The Netherlands; Public Health Laboratory, GGD Amsterdam, Cluster Infectious Diseases, Nieuwe Achtergracht 100, Amsterdam, 1018 WT The Netherlands

**Keywords:** Hepatitis B virus, Genotype, MSM, HIV-1

## Abstract

**Background:**

Hepatitis B virus (HBV) variants belong to different genotypes, A-J, whose worldwide distribution is linked with geography, probably because viral spread was associated with ancient human migrations. HBV genotype G (HBV-G) is an aberrant genotype with little sequence divergence, suggesting a recent origin. HBV-G is strongly associated with certain risk groups such as intravenous drug users (IDUs) and men who have sex with men (MSM), but hardly with geography. The origin and epidemiology of HBV-G remain unresolved, as is the disease association.

**Methods:**

To estimate the prevalence and possible time of introduction of HBV-G into the MSM community in Amsterdam, the Netherlands, we have retrospectively analysed 226 blood serum samples from HBsAg positive MSM enrolled in the Amsterdam Cohort Studies (ACS) on HIV infection and AIDS dating from 1984 to 1999 using genotype-specific PCR assays.

**Results:**

Of the 226 HBsAg-positive samples, 149 were HBV DNA positive. Of those, 104 were positive for HBV genotype A (HBV-A) and five for HBV-G, and 40 showed a dual infection with both HBV-A and HBV-G. Being HIV-infected was significantly associated with a reduced HBV DNA viral load in blood, but not with the prevalence of HBV-G. Early virus already contained stop codons in the precore region and a 36 bp insertion in the core gene which are the characteristics of HBV-G.

**Conclusions:**

HBV-G was introduced before 1985 into the Amsterdam MSM community. Early isolates show very limited sequence variation, confirming a low evolutionary rate. HBV-G acquisition was independent of HIV infection, but being HIV-infected was significantly associated with a reduced HBV viral load in blood, indicating a beneficial effect of early HIV infection in controlling HBV replication.

**Electronic supplementary material:**

The online version of this article (doi:10.1186/s12879-016-1599-7) contains supplementary material, which is available to authorized users.

## Background

The hepadnavirus hepatitis B virus (HBV), a causative agent of cirrhosis and liver cancer, is currently divided into ten genotypes, named A-J, several subgenotypes and recombinant forms whose dispersal is largely connected with geography [1]. For instance, genotype A is the most common variant in northern Europe, while genotype D is more prevalent in eastern and southern Europe [1]. Genotype G, an aberrant HBV genotype without a clear geographic association, is also present in certain risk groups in Europe. The prevalence of HBV infection is low in western Europe, e.g. HBsAg prevalence is <1 % in most northern and western countries, and <0.5 % in the Netherlands [2]. Higher rates of HBV infection in Europe are apparent for migrants, men who have sex with men (MSM) and intravenous drug users (IDUs). HBV-A2 is the most prevalent HBV genotype in the Netherlands, in particular among MSM [3]. Infections with HBV-G occur in this risk group, but interestingly mainly as co-infections with HBV-A2 and human immunodeficiency virus type 1 (HIV-1) [4]. Worldwide, HBV-G has almost exclusively been found in HIV-infected MSM and IDUs [5–12], suggesting a strong association with those risk groups. Although HBV-G was also detected in two Mexican children, the genotype is not endemic in Mexico and the children were most likely infected either through blood transfusion or close contact with a family member that might belong to a risk group [13]. Mono-infections with HBV-G are uncommon [14–16] as the virus apparently does not replicate well due to a 12 amino acid insertion in the Core protein that interferes with virion secretion and stop codons in the precore region that prohibit HBeAg expression [17, 18].

Rescue by a helper virus restores HBV-G production such that it can even outcompete the helper genotype [19–21]. Most HBV-G infections described are thus co-infections with HBV genotype A in France [22], Spain [6], Japan [7, 20], the USA [21, 23] and Canada [9], with HBV genotypes A, C or D in Germany [24, 25], with HBV genotype A or H in Mexico [8], and with HBV genotype F in Argentina and Brazil [11].

Global HBV-G isolates are closely related and show very little sequence divergence [26], suggestive of a relatively recent evolutionary origin [27], and/or a comparatively recent introduction in the risk groups. It is thus possible that HBV-G was a recent introduction into the Netherlands. Retrospective incidence data and sequence analysis of early virus isolates could thus shed light on the history of this peculiar HBV-G genotype. The Amsterdam Cohort Studies (ACS) on HIV and AIDS started in October 1984 with the inclusion of MSM and IDUs from Amsterdam, the Netherlands [28]. The early blood samples were collected before the era of effective and extensive HBV vaccination and antiviral treatment. Therefore, we set out to retrospectively determine the prevalence of HBV-G and HBV-A infections in HBsAg- positive participants of the ACS with or without HIV infection that were collected from 1984 to 1999. In addition, a characterization of certain genome characteristics was performed for historical HBV-G isolates.

## Methods

### Patient material

The ACS started enrolling asymptomatic 18–65 year old MSM with at least two sexual partners in the 6 months prior to intake in October 1984. Participants were mainly recruited through the gay press, advertisements and by word of mouth. An undetermined number had taken part in an earlier trial investigating the efficacy of an HBV plasma-derived vaccine that was running from November 1980 till December 1981 [28]. Between April 1985 and February 1988 only HIV-seronegative men could enter the study. From February 1988 until December 1998, admission was re-opened for HIV-infected individuals.

From the ACS, blood samples from MSM were selected that met the following criteria: HBsAg-positive and preferably also HBcAb-positive and sampled before the year 2000 [29]. A total of 226 patient samples were HBsAg-positive, of which 222 also tested HBcAb-positive at the same moment. CD4+ T-cell counts at sampling date were available for 196 participants, both HIV-positive and HIV-negative MSM. All blood samples have been stored at −80 °C for the entire period; thaw-freeze cycles were limited to 0–1 during storage.

### Real-time PCR assays

Viral DNA was isolated from serum samples with the QIAamp UltraSens Virus Kit (QIAGEN, the Netherlands). Single-tube real-time PCR reactions that specifically quantify HBV genotype A or G DNA were performed as described [4], for the primer sequences, see also Additional file 1: Table S1. The primers and probes used are highly specific for the targeted HBV genotype (either A or G) and have a lower limit of detection of 5 copies per reaction (=1000 copies/ml) of the targeted genotype in a background of up to 10^8^ copies of the non-targeted genotype [4].

### Genotype-specific PCR assays

Real-time PCR results were confirmed using a genotype A or G specific nested PCR. The first PCR spans the 3’end of the pol gene and part of the X gene (nt 692–1823 from the EcoRI site). The nested PCR amplifies a fragment of 762 base pair (bp) (nt 902–1664 from the EcoRI site), and is able to detect 5–10 copies of input DNA.

Genotype-specific primers amplifying the precore region were developed for HBV-C, D, F and H with the first PCR amplifying a fragment spanning nt. 1728–2071 and a nested PCR amplifying nt 1751–2034. All genotype-specific primer sequences are given in Additional file 1: Table S1. The nested PCR’s detect 5–10 copies of the specific HBV-DNA per reaction. Any amplified products were analysed by sequencing (see below). Non-target genotypes were sometimes amplified when the input DNA of the non-targeted genotype was ≥10^5^ copies/ml.

To investigate the presence of stop codons in the precore gene and the 36 bp insertion in the core gene, a fragment of 232 (HBV-A) or 268 (HBV-G) bp, encompassing the precore and core region of the viral genome (see Additional file 1: Table S1 for primer sequences), was amplified with a nested PCR that detects 5–10 copies of HBV-DNA per reaction, from blood serum samples and directly sequenced with the BigDye Terminator cycle sequencing kit (Applied Biosystems, Foster City, CA, USA). Electrophoresis and data collection were performed on an ABI PRISM 3100 genetic analyser (Applied Biosystems, Foster City, CA, USA). Sequences were assembled with CodonCode Aligner [30], and aligned with reference HBV sequences from the NCBI nucleotide database [31] using ClustalW implemented in BioEdit Sequence Alignment Editor version 7.0.9 [32].

### HBeAg analysis

HBeAg expression in apparently HBV-G mono-infected samples was analysed using the Liaison® HBeAg chemiluminescence immunoassay (DiaSorin SpA, Saluggia, Italy).

### Statistical analysis

Statistical analyses were performed in GraphPad Prism 5 (GraphPad Software, La Jolla, CA).

## Results

### Prevalence of HBV-G in MSM in Amsterdam 1984–1999

A total of 226 serum samples from the ACS that were HBsAg-positive and in majority also HBcAb-positive were retrospectively analysed for the presence of HBV-A and/or HBV-G DNA using real-time PCR assays and a confirmatory PCR with genotype-specific primers.

**Fig. 1:**
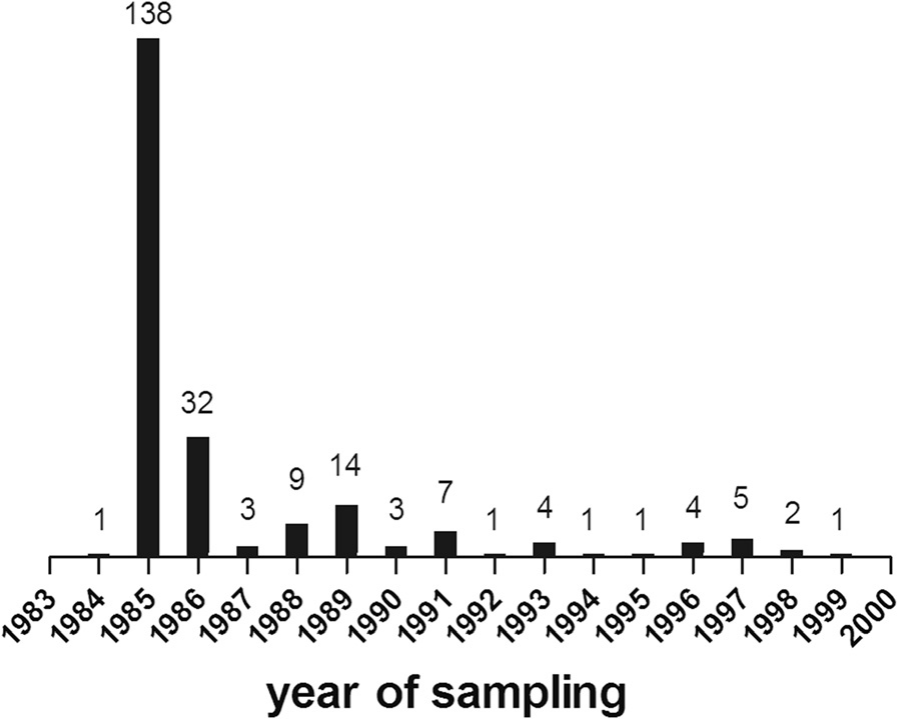
Number of Dutch MSM samples selected from the ACS with regard to year of sampling. Numbers of blood serum samples selected from the ACS are shown. Samples were selected when they fulfilled the following criteria: originating between 1984 (start of the ACS) and 1999, and having been tested HBsAg and preferably HBcAb positive

The majority of samples selected were from 1985 to 1986 (170/226 samples, 75 %) (Fig. 1). A total of 149/226 (66 %) of the samples were HBV DNA positive (Table 1), and of those, 40 samples (27 %) displayed a dual infection with both HBV genotypes A and G, while five sample appeared to be mono-infected with HBV-G. Amplification of other “helper” HBV genotypes from these samples remained negative with both type-specific and more general primers.

**Table 1:** HBV-A and HBV-G DNA detection in blood serum samples from Dutch MSM 1984-1999

HBV genotype amplified	No. of samples	No. of HIV-positive samples	No. of HIV-negative samples
	*N* = 226 (100 %)	*N* = 152	*N* = 74
HBV-A	104	61	43
HBV-G	5	3	2
HBV-A and -G	40	31	9
*Total no. of samples with HBV-A and/or HBV-G DNA*	*149 (66 %)*	*95 (63 %)*	*54 (73 %)*

In this cross-sectional analysis, HBV DNA prevalence was slightly higher (73 %) in HIV-negative MSM than in HIV-positive MSM (63 %), but this difference was not statistically significant (Chi-square test *p* > 0.1). Dual infections with both HBV-A and HBV-G were more prevalent in HIV-positive MSM (33 % vs. 17 % in HIV-negative MSM) but this difference was again not significant (Chi-square test *p *> 0.05). Mono-infections with HBV-G were equally distributed between the two groups: 2 mono-infections (4 %) with HBV-G in HIV-negative versus 3 mono-infections (3 %) in HIV-positive MSM.

**Fig. 2:**
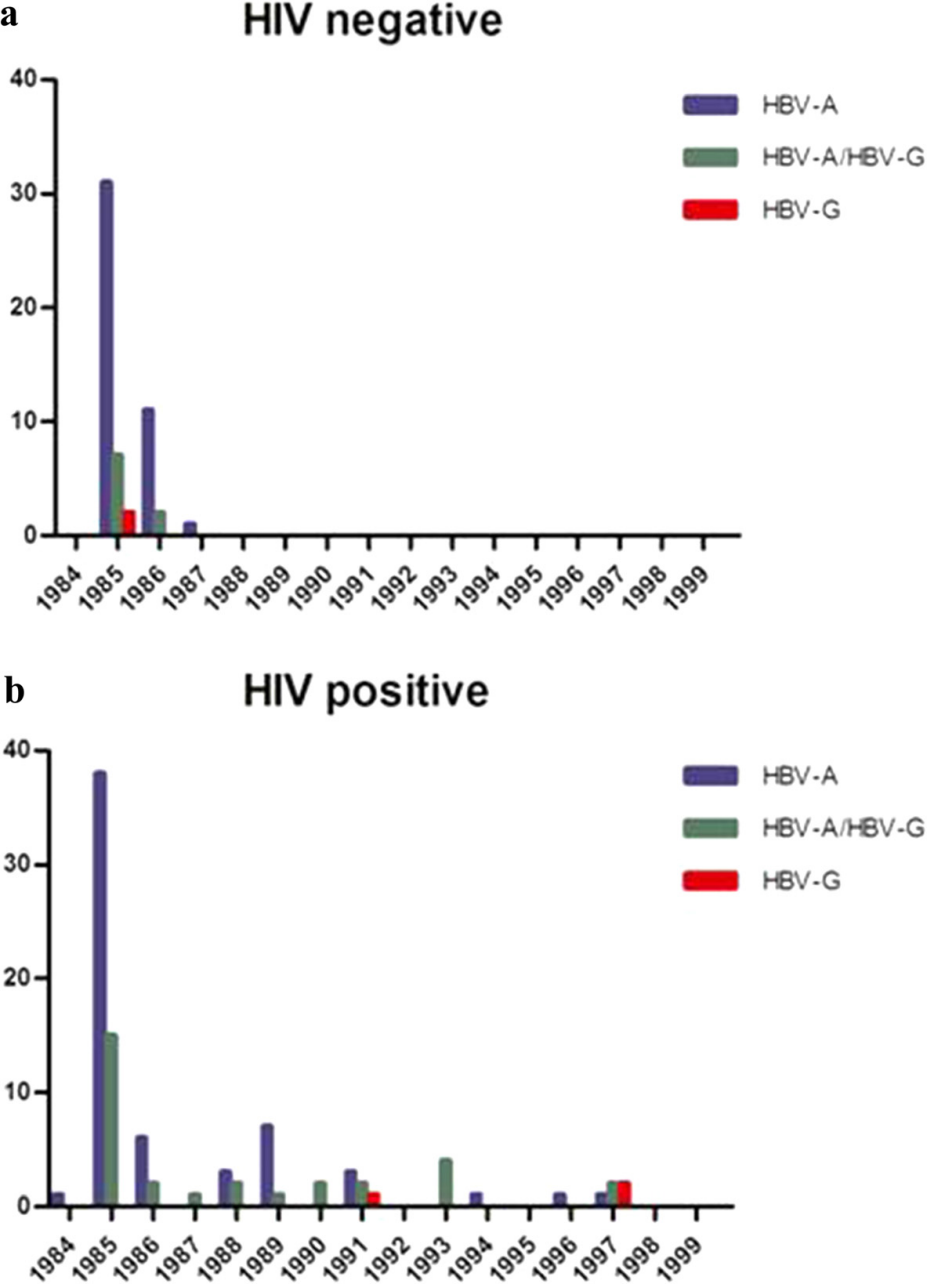
Numbers of MSM that were HBV-A and/or HBV-G DNA positive with regard to year of sampling and HIV status. The numbers of MSM from the ACS during 1984–1999 that tested either HBV-A, HBV-G or HBV-A and HBV-G positive using two genotype-specific real-time PCR assays followed by a genotype-specific nested PCR to confirm the real-time PCR results. Results are shown in panel (**a**) for HIV-negative MSM (*N* = 74) and in panel (**b**) (*N* = 95) for HIV-positive MSM

Surprisingly, HBV-A and HBV-G DNA was only present in samples from 1985 to 1987 in HIV-negative MSM, but not in subsequent years (Fig. 2a). For HIV-positive MSM, HBV DNA could be detected in samples up to 1997, although the numbers of positive samples decreased over time (Fig. 2b). These findings correlate well with the strongly declining numbers of individuals after 1986 that met our selection criteria involving serological evidence of HBV infection (Fig. 1), and are in line with an earlier report on a profound decline of the HBV incidence in the ACS after the first years of its establishment [29].

### Prevalence of HBV-G in MSM in Amsterdam in 1985 with regard to their HIV status

Because HBV DNA prevalence decreases sharply after 1986 in HIV-negative MSM, the prevalence of HBV DNA in blood was calculated for 1985, the year with the highest number of HBV positive MSM, to compare values between those infected and uninfected with HIV (Fig. 2). In 1985, HBV-G DNA was detected in 9/40 (23 %) of the HBV DNA positive/HIV-negative MSM and in 15/53 (28 %) of the HBV DNA positive/HIV-positive MSM (Fisher’s exact test, two-tailed, *p* > 0.1), implicating that HBV-G infection was not more common among those infected with HIV than those without HIV infection. Adding data for 1986, the only other year with appreciable HBV DNA positivity for HIV-uninfected men, did yield similar prevalence values for HBV-G infection (21 % in HIV- uninfected versus 28 % in HIV- infected MSM, Fisher’s exact test, two-tailed, *p* > 0.1).

### HBV-G mono-infection and HBeAg expression

HBV-G does not express Hepatitis B “e” antigen (HBeAg), probably due to its non-functional precore region [14, 15, 17]. So, in any HBV-G positive samples displaying HBeAg positivity, expression is most likely due to co-infection with another HBV genotype. Therefore, we measured HBeAg in the five apparent HBV-G mono-infections, and found it to be present in one of these five samples (Table 2). Unfortunately, in the three samples from 1985 to 1991, dual infected samples used as controls were also HBeAg-negative, suggesting that sample age and/or storage conditions could have an effect on the assay, limiting its predictability. Alternatively, samples could be HBeAg-negative due to preceding HBeAb seroconversion. From the four samples tested from 1997, the dual infected samples and one of the apparently mono-infected samples (no. ACS-970660) exhibited HBeAg positivity, whereas the other mono-infected sample (no. ACS-971256) was negative for HBeAg, suggesting that it could represent a true HBV-G mono-infection. Repeated attempts to amplify a helper HBV genotype from samples ACS-850676, ACS-850530, ACS-916078, ACS- 971256 that were HBeAg negative and sample ACS-970660 that was HBeAg-positive, failed. Using other samples from patient ACS-970660 collected 14 days before and 29 days after sample ACS-970660 was obtained also did not result in the amplification of another HBV genotype. Using forward and reverse primers destined for specific genotypes in various combinations also did not result in the amplification of PCR products of the expected lengths for any of the samples.

**Table 2:** HBeAg in HBV-G mono-infection

Patient no.	Year	HIV serology	HBV genotype PCR	HBV-A cps/ml	HBV-G cps/ml	HBeAg (PEI U/mL^a^)	HBeAg result
ACS-850676	1985	NEG	G	---	2.69E + 02	<0.0100	NEG
ACS-850530	1985	NEG	G	---	1.67E + 02	<0.0100	NEG
ACS-916078	1991	POS	G	---	1.39E + 02	<0.0100	NEG
ACS-971256	1997	POS	G	---	3.14E + 04	<0.0100	NEG
ACS-970660	1997	POS	G	---	9.49E + 09	3.61	POS
ACS-850622	1985	NEG	A/G	5.83E + 05	5.52E + 05	<0.0100	NEG
ACS-850626	1985	NEG	A/G	1.44E + 05	3.85E + 03	<0.0100	NEG
ACS-916084	1991	POS	A/G	7.22E + 03	8.01E + 02	<0.0100	NEG
ACS-972833	1997	POS	A/G	5.59E + 08	2.36E + 08	7.57	POS
ACS-972876	1997	POS	A/G	1.65E + 08	3.34E + 02	13.5	POS

^a^Paul Ehrlich Institute units/mL

### HBV-G and HBV-A serum viral load in HIV-positive and HIV-negative individuals

As HBV DNA detection was performed using a real-time PCR assay, the viral load of the samples could be calculated. HBV-A, acting as a helper virus, is known to increase the HBV-G viral load, therefore, the HBV-G viral loads in mono- or dual infected samples should be analysed separately. However, mono-infection with HBV-G is a rare phenomenon, which was also the case in the present study. Therefore, we only analysed the HBV-G viral load in HBV-A and HBV-G double-infected MSM. For HIV-negative MSM, only samples from 1985 to 1986 yielded HBV DNA, therefore the HBV-G viral load comparisons were restricted to that period (Table 3). A log_10_ lower mean HBV-G viral load was found in HIV-infected MSM, which was statistically significant (Student’s *t*-test, two-tailed, *p* = 0.0063).

**Table 3:** HBV-A and HBV-G viral load in HBV double- or mono-infected MSM from 1985 to 1986 with or without HIV infection

1985-1986	N	HBV-A/G infected	HBV-A/G infected	N	HBV-A mono- infected
		Mean HBV-G viral load log_10_ (range)	Mean HBV-A viral load log_10_ (range)		Mean HBV-A viral load log_10_ (range)
HIV- negative samples	9	4.06 (2.96–5.74)	5.32 (3.73–8.76)	42	5.66 (1.30–9.46)
HIV- positive samples	17	2.94 (0.62–4.49)	3.69 (2.18–4.80)	44	4.53 (2.20–9.66)
*p*-value*		0.0063	0.0008		0.040

*Student’s *t*-test, two-tailed

The HBV-A viral load was calculated from the real-time PCR data for four groups of patients in 1985 and 1986 (*N* = 112): those that were HIV-positive and either HBV-A mono-infected (*N* = 44), or HBV-A and -G dual infected (*N* = 17), as well as for these two groups without HIV infection (*N* = 42 and *N* = 9, respectively). The mean HBV-A viral load was found to be significantly lower in the group that carried all three viruses (HIV, HBV-A and HBV-G) compared with the HIV-negative group that was infected with HBV-A and HBV-G (Table 3, Student’s *t*-test, two-tailed, *p* = 0.0008). Comparing the HIV-positive and negative groups that were infected with HBV-A alone also showed a significant difference between their mean HBV-A viral load (Student’s *t*-test, two-tailed, *p* = 0.04).

A remarkable observation is that in the triple infected group, a viral load in the higher range (>10^5^ copies/ml) was never measured in the 17 patients analysed, whereas a viral load between 10^5^ and 10^9^ copies/ml was frequently seen in those infected with HBV-A alone, irrespective of their HIV status (not shown). In fact, in 12/44 (27 %) of the HIV-positives and 19/42 (45 %) and 5/9 (56 %) of the HIV-negatives infected with HBV-A or HBV-A and -G, respectively, the viral load was between 10^5^ and 10^9^ copies/ml. An extremely high viral load (>10^9^ copies/ml) was only observed in persons infected with HBV-A without HBV-G, irrespective of their HIV status. Of the HIV-positives, 6/44 (14 %) and of the HIV-negative individuals 12/42 (29 %) carried a viral load >10^9^ copies/ml.

### Correlation of HBV-A and HBV-G serum viral loads

**Fig. 3:**
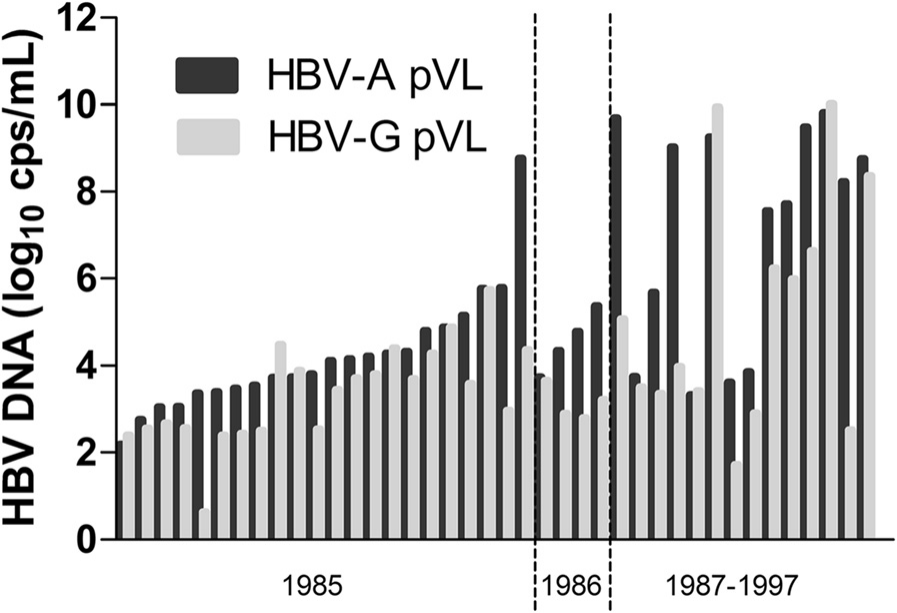
HBV-A and HBV-G serum viral load in dual infected individuals. Log_10_ transformed HBV serum viral loads, as determined using a two genotype-specific real-time PCR assays, are shown for 40 Dutch MSM who were dually infected with HBV-A/HBV-G, irrespective of HIV status, during 1985–1997. After 1986, all dually infected MSM were HIV-positive

The HBV-A and HBV-G viral load in dual infected individuals (*n* = 40) is depicted in Fig. 3. In half of the cases, viral loads for both genotypes are very similar with 20/40 (50 %) samples showing less than one log_10_ difference in viral load. The HBV-A viral load was significantly higher than the HBV-G viral load in the other half of cases. In no cases did the HBV-G viral load exceed the HBV-A viral load by more than one log_10_ difference, although there were two cases where the HBV-G viral load was slightly higher than that of HBV-A. Both latter samples were from HIV-positive individuals.

### CD4+ T cell counts and HBV-DNA in HIV-positive individuals

**Fig. 4:**
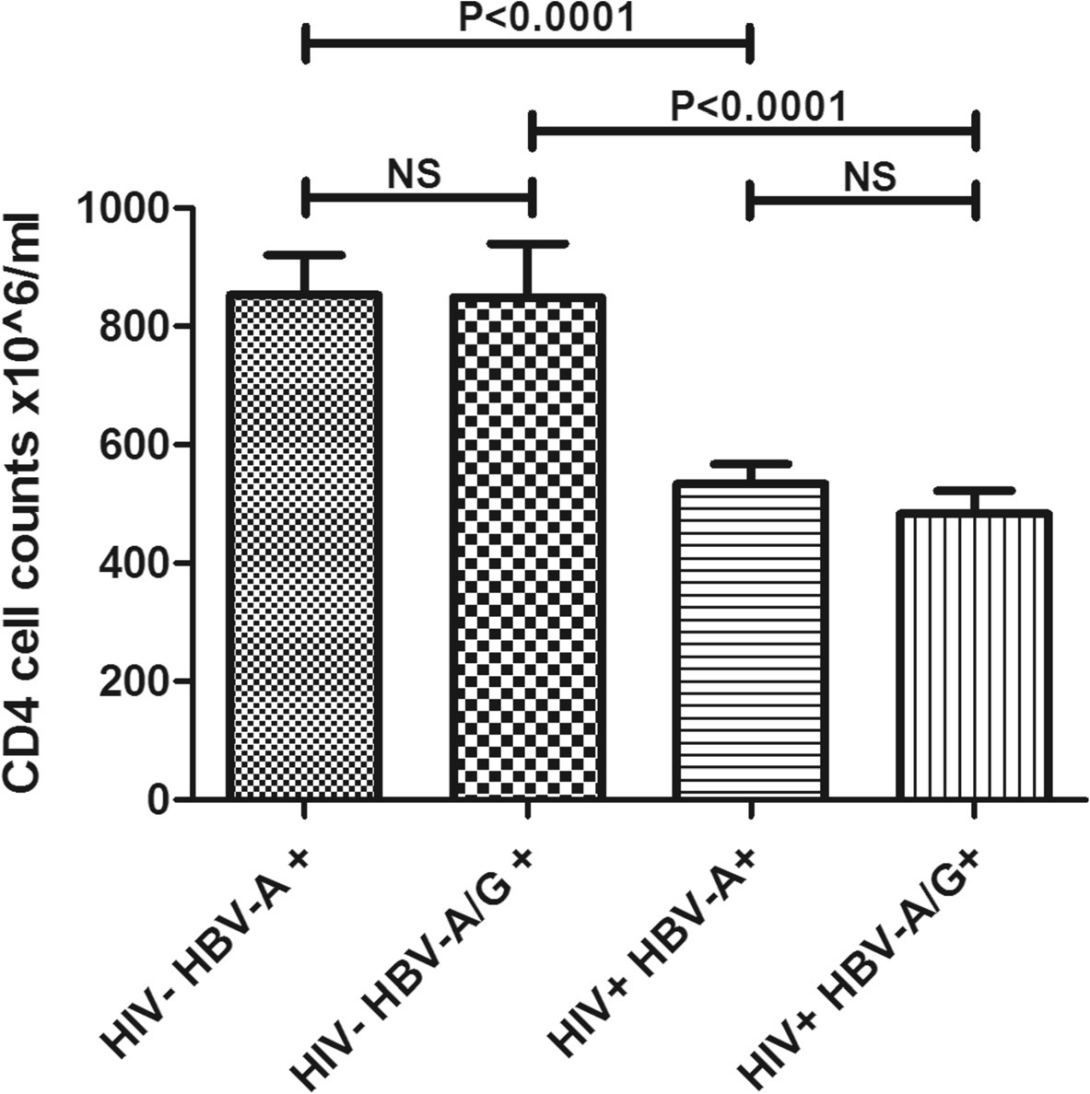
Mean CD4+ T-cell counts in ACS participants with or without HIV infection and measurable HBV-A or HBV-A/HBV-G DNA. The mean CD4+ T-cell counts are shown for HIV-negative/HBV-A DNA positive MSM (*N* = 30, mean 853 ± 68 cells/μl), HIV-negative/HBV-A + HBV-G DNA positive MSM (*N* = 9, mean 848 ± 90 cells/μl), HIV-positive/HBV-A DNA positive MSM (*N* = 57, mean 534 ± 34 cells/μl), and HIV-positive/HBV-A + HBV-G DNA positive MSM (*N* = 28, mean 483 ± 39 cells/μl), respectively. Statistical significance is indicated by p-values (Student’s *t*-test, two-tailed, NS = not significant). CD4+ T-cell ranges are 290–1710 cells/μl for HIV-negative MSM and 90–1310 cells/μl for HIV-positive MSM

For 39 ACS participants without HIV infection and 96 participants with HIV infection and measurable HBV DNA, CD4+ T-cell counts at sampling date were available. Comparison of the mean CD4+ T-cell count between HIV-negative and HIV-positive individuals showed a large difference of around 300 cells/μl, which was statistically significant (Student’s *t*-test, two-tailed, *p* < 0.0001) (Fig. 4), as would be expected. There were no significant CD4+ T-cell count differences with regard to single (HBV-A) or dual infection (HBV-A + HBV-G), both in the HIV-negative or positive groups (Fig. 4). Also, there was no significant correlation between the HBV viral load in mono versus dual infected HIV-positive participants (Spearman’s rank correlation coefficient r_s_ = −0.06, *p* = 0.66), suggesting that - at least in this cohort-, CD4+ T-cell numbers are not related to HBV replication level. Analysing samples from the early years (1985–1986) of the ACS separately from later years (1990–1997) did not change these results.

### Molecular characteristics of HBV-G during 1985–1999: a G1776A mutation in a subset of patients

**Fig. 5:**
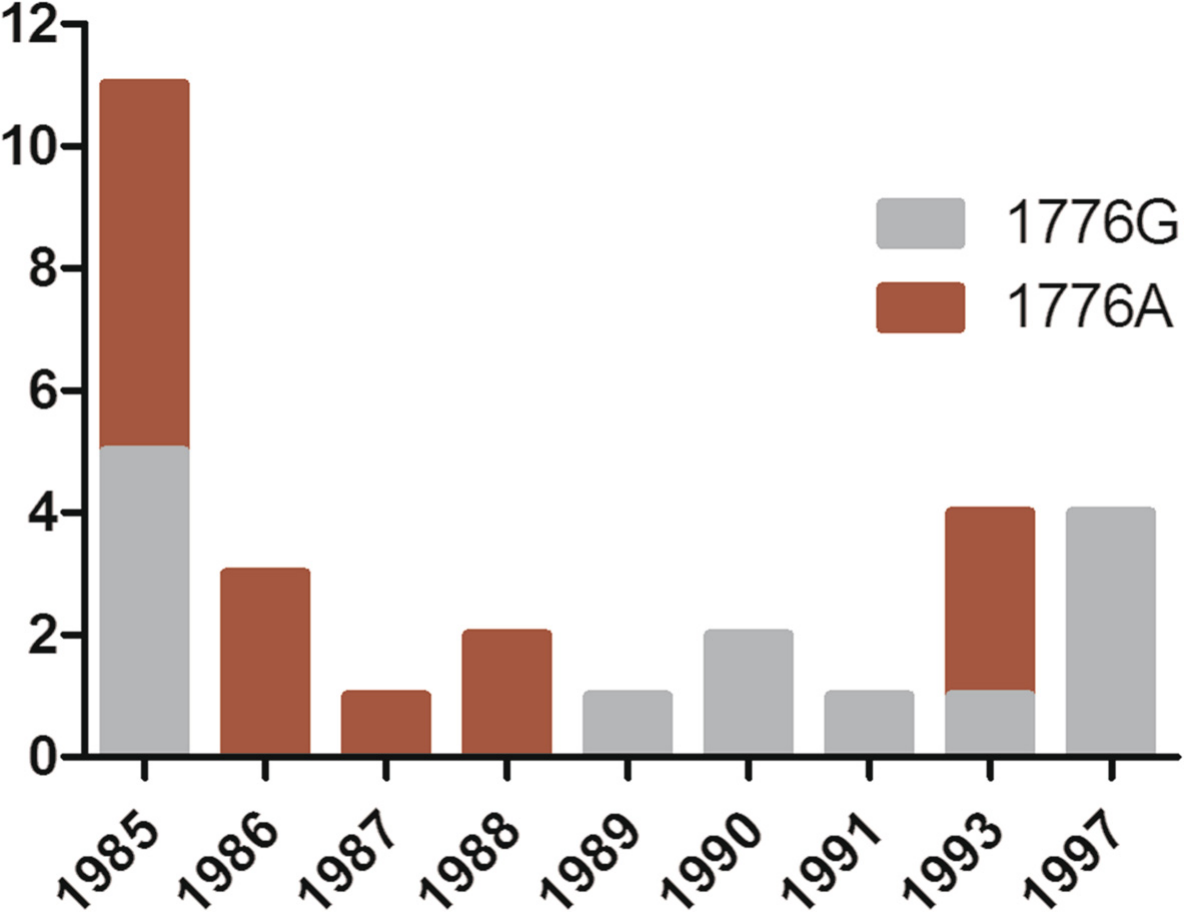
Numbers of HBV-G isolates with or without the G1776A mutation during 1985–1997. A nested PCR amplifying nt 1751–2034 of the HBV genome was sequenced for 29 HBV-G isolates to investigate the evolution of this region during 1985–1997. A G1776A mutation was detected in 15 of the isolates dating to 1985–1993

For 29 samples from 45 HBV-G infected individuals, sufficient quantities of the blood serum sample were available to amplify a 268 bp fragment spanning the precore and core region of the genome for sequence analysis. The sequences were investigated for the presence of the characteristic features of present-day HBV-G, one or two stop codons in the precore region and a 36 bp insertion into the core gene. All sequences from 1985 till 1997 contained these features, suggesting that HBV-G from 1985 was genetically similar to the later isolates. The only sequence variation observed was a single G1776A mutation that was present in 15 HBV-isolates from 1985 to 1997 (Fig. 5). Six HBV-G isolates carrying this mutation were from HIV-negative MSM and 9 HBV-G isolates were from HIV-positive MSM.

## Discussion

In this study, we have retrospectively analysed the prevalence of HBV genotype G in MSM from the Amsterdam area in the early years of the HIV epidemic and found it to be widely present from 1985 onwards in HBV-seropositive MSM. The early HBV-G isolates were genotypically highly similar to later isolates and contained the distinctive characteristics of HBV-G, being two stop codons in the precore region precluding HBeAg expression and a 36 bp insertion in the core gene. In 1985 and 1986, the prevalence of HBV-G was similar between HIV-positive and HIV-negative MSM, suggesting that risk group and thus risk behaviour is important for acquisition of the virus. Being HIV- infected apparently does not serve as an additional risk factor. After 1986 there was a sharp decrease in the HBV DNA positivity that was already observed as a decline in seroprevalence during patient selection for the present study. The steep decline in HBV prevalence has been observed for the ACS in general. Van Houdt et al. reported that the overall incidence of anti-HBc seroconversion was highest in 1985–1986, declined afterwards, and remained stable thereafter up to 2003, when routine vaccination was started for this risk group [29]. HBV-DNA positivity in HIV-negative MSM disappeared after 1987, although two HIV-negative samples from that period (dating to 1996 and 1999, respectively) were selected for this study because of positive HBV serology. The decline in HBV positivity possibly relates to decreased risk behaviour as anti-HBV vaccination was negligible at that time. It is possible that the age of the samples could have influenced our study, as some samples have been stored for almost 30 years. However, we believe that sample age did not influence our results, firstly because most samples that were HBV DNA positive were from 1985, and secondly because the real-time PCR amplifies very short DNA fragments, and in general only amplification of longer fragments is affected by DNA degradation.

HBV-G sequence variation in the 1985–1997 period was extremely low. Two fragments of in total 1029 bp of the 3.2 kb genome were sequenced for 29 Dutch HBV-G isolates and only a single nucleotide substitution, G1776A, was found in 50 % of the isolates from 1985 to 1993 in both HIV-negative and positive individuals. This specific mutation is often seen in the chronic phase of other HBV genotypes, which have been correlated to liver pathology [17, 33]; it has not yet been reported in HBV-G sequences obtained from other geographic regions than the Netherlands. The G1776A mutation contributes to the “late stage” genotype of HBV-G that also includes the 2 precore stop codons and the T1753C/A1762T/G1764A/G1896A core promoter mutations that are fixed in HBV-G genomes. HBV-G infection has been associated with liver fibrosis [10, 22], although others found no such relation [34]. In mice, dual, but not single infection with HBV-A and HBV-G or HBV-H and HBV-G leads to increased liver fibrosis [35, 36]. It is possible that the G1776A mutation occurred de novo in different patients, but it seems more likely that these two strains were circulating in Amsterdam at least since 1985. An HBV-G strain from an HIV-positive patient presenting with acute HBV-A and -G infection in Amsterdam in 2003 also did carry this mutation [4], suggesting that the variant strain did not disappear from the Amsterdam region after 1993.

The low sequence variation seen in HBV-G during 1985–1997 is not unique for this specific genotype, as very little nucleotide differences were reported for a 672 nt pre-S2 and S region fragment of HBV-A in the same cohort from 1985 till 2002 [29]. A recent phylogenetic analysis indeed proposed a very low substitution rate for HBV [27].

Five apparent HBV-G mono-infections were detected in MSM samples from 1985, 1991 and 1997. No other genotype commonly associated with HBV-G infection (e.g. HBV-A, −C, −D, −F, or -H) could be amplified from these samples. All five samples were HBsAg positive, which is not incompatible with HBV-G mono-infection. HBsAg expression can be decreased or delayed in mono-G infections, but it has frequently been reported to occur [14–16]. HBV-G apparently does not express HBeAg [17], and therefore the samples were tested for the presence of the HBV “e” antigen. Indeed, four samples were HBeAg-negative, which could be an indication, but not definite proof, of mono-infection. In HBeAg-positive infection, expression leads to HBeAb seroconversion and elimination of hepatocytes expressing HBeAg, and thus to disappearance of detectable HBeAg (see: [37]). Acute HIV-1 infection can lead to a loss of HBeAg, but none of our five patients were HIV seroconverters at the time of sampling, and two individuals were actually HIV-negative. Control dual infected samples from 1985 and 1991 also showed no measurable HBeAg levels, suggesting that sample age and/or storage condition could have interfered with performance of the assay. Summarizing, only one sample from 1997 could likely represent an HBV-G mono-infection, as HBeAg expression in the other sample from 1997 suggests the presence of a helper genotype. However, repeated attempts to amplify this virus failed, even when using patient samples from an earlier or later time point.

A cross-sectional analysis of the viral load of HBV-A and HBV-G in MSM from the early years of the HIV epidemic in Amsterdam revealed that in 1985–1986, being HIV-infected resulted in a significantly lower viral load of both HBV-A and HBV-G compared with HIV-uninfected MSM. It has been reported that acute HIV-1 infection results in decreased HBV DNA levels, suggesting that the immune activation induced by HIV is beneficial for controlling HBV replication [38, 39]. In some cases, the HBV DNA viral load remained low for as long as two years after acute HIV-1 infection [38]. In the ACS, MSM enrolled in 1985–1986 were not likely to be in the AIDS stage of HIV infection as they had to be asymptomatic to be eligible for inclusion. This may provide an explanation for the significantly decreased HBV viral load in the HIV-positive group. In this cohort, absolute CD4+ T-cell counts were not related to HBV viral load, neither in mono-infected nor in dual HBV-infected individuals. This suggests that CD4+ T-cells are not significantly involved in suppression of HBV replication. In a large multinational study of HBV and HIV-infected individuals, low CD4+ T-cell counts were related to high HBV DNA loads [40]. However, the relation was best noticeable when CD4+ T-cell counts declined to below 50 cells/μl, which is much lower than the CD4+ T-cell counts in our patient selection. In addition, in MSM with chronic HBV-infection an association between HIV infection and higher HBV levels has been reported [41]. As the chronicity of HBV infection has not been determined in our cohort, e.g. liver enzymes and liver pathology were not investigated, this discrepant finding might be related to the heterogeneous composition of our selection with regard to the unidentified stages of both HBV and HIV infections.

## Conclusions

An analysis of the prevalence and characteristics of HBV genotype G in 1984–1999 in a cohort of MSM in the Netherlands was performed in the present study. The results show that HBV-G was already widely present among Dutch MSM in 1985. In this cohort, HIV status was not associated with HBV-G infection, but the HBV viral load of both genotype A and G in mono-A or dual A/G infected MSM was significantly lower in HIV-infected men, confirming the beneficial effect of HIV infection in controlling HBV replication. HBV-G isolates from 1985 contain both genetic features reported for this genotype: stop codons in the precore region and a 36 bp insertion in the core gene. HBV-G sequence variation was extremely limited to a G1776A mutation that was seen throughout the study period in approximately half of the patient isolates, suggesting the circulation of two slightly divergent HBV-G lineages among MSM in the Netherlands.

## Abbreviations

HBV, hepatitis B virus; HIV, human immunodeficiency virus; IUD, intravenous drug use; MSM, men who have sex with men; nt, nucleotide; bp, base pair

## Additional file

Additional file 1: Table S1. Contains the sequences of the PCR-primers used in the study. (XLS 39 kb)
